# Bit1 knockdown contributes to growth suppression as well as the decreases of migration and invasion abilities in esophageal squamous cell carcinoma via suppressing FAK-paxillin pathway

**DOI:** 10.1186/s12943-016-0507-5

**Published:** 2016-03-08

**Authors:** Tianli Fan, Jing Chen, Lirong Zhang, Pan Gao, Yiran Hui, Peirong Xu, Xiaqing Zhang, Hongtao Liu

**Affiliations:** Department of Pharmacology, School of Basic Medicine, Zhengzhou University, Zhengzhou, Henan 450001 P.R. China; Department of Oncology, the Zhengzhou Central Hospital Affiliated to Zhengzhou University, Zhengzhou, Henan 450007 P.R. China; School of Pharmaceutical Sciences, Zhengzhou University, Zhengzhou, Henan 450001 P.R. China; Laboratory for Cell Biology, College of Life Sciences of Zhengzhou University, Zhengzhou, Henan 450001 P.R. China

**Keywords:** Bcl-2 inhibitor of transcription 1, FAK-paxillin pathway, esophageal squamous cell carcinoma, cell migration, cell invasion

## Abstract

**Background:**

There is growing evidence that Bit1 exerts different roles in the development and progression of human cancers. Although Bit1 was highly exhibited in ESCC tissues in our previous study, its roles and molecular mechanisms implicated in development and progression of ESCC remain unknown.

**Methods:**

Bit1 protein expression in ESCC cell lines and normal esophageal epithelial cell was detected by Western blotting. Bit1 protein expression mediated by Bit1 shRNA was investigated by Western blotting. MTT, migration assay, invasion experiment, ELISA and Flow cytometry were utilized to determine the effects of Bit1 knockdown on cell proliferation, migration, invasion and apoptosis, respectively. A xenograft model was used to examine in vivo tumourigenicity, and immunohistochemistry and TUNEL were utilized to evaluate the related protein expression and apoptosis. Gene microarray was determined by Agilent SurePrint G3 Human GE 8 × 60 K Microarray, the interaction of Bit1 and FAK proteins were detected by Immunoprecipitation and the key protein expressions of FAK-paxillin pathway were detected by Western blotting.

**Results:**

We found Bit1 expression in all human ESCC cell lines tested was significantly higher than that in normal esophageal epithelial cell Het-1A (*P* < 0.05), in which EC9706 presented the highest Bit1 level. Bit1 protein level was significantly downregulated at day 1 after transfection with specific shRNA against Bit1 (*P* < 0.05). At days 2 and 3, Bit1 level reached the lowest value after transfection with Bit1 shRNA. Moreover, Bit1 depletion contributed to growth inhibition *in vitro* and *in vivo*, reduced cell migration and invasion abilities, and induced cell apoptosis in EC9706 and TE1 cells. More importantly, Bit1 downregulation significantly lowered Bcl-2 and MMP-2 levels in EC9706 xenografted tumor tissues, meanwhile triggered apoptosis after treatment with different doses of Bit1 shRNA. Further gene microarray revealed that 23 genes in Bit1-RNAi group were markedly downregulated, whereas 16 genes were obviously upregulated. Notably, Bit1 intrinsically interacted with FAK protein in EC9706 cells. Moreover, paxillin was downregulated at mRNA and protein levels in Bit1 shRNA group, coupled with the decreases of FAK mRNA and protein expressions.

**Conclusion:**

Bit1 may be an important regulator in cell growth, apoptosis, migration and invasion of ESCC via targeting FAK-paxillin pathway, and thereby combinative manipulation of Bit1 and FAK-paxillin pathway may be the novel and promising therapeutic targets for the patients with ESCC.

## Background

Esophageal cancer is the one of the most frequently occurring malignant neoplasms worldwide [1, 2], which consists of two main subtypes, namely esophageal adenocarcinoma (ECA) that is more common in the developed countries, such as North America and Europe [3, 4], and esophageal squamous cell carcinoma (ESCC) that is frequently occurring in the developing countries [4–6], such as East Asian countries. At present, ESCC is still the main histological subtype, accounting for more than 90 % of cases of all esophageal cancers, with the less than 10 % in 5-year survival rate of the patients with ESCC [7–9]. Most importantly, about 50 % of the patients with ESCC exhibit the local invasion and metastasis at the time of diagnosis, which will lead to the high mortality [10, 11]. Despite recent advances in therapeutic strategies, a large number of patients with ESCC remain poor clinical outcome. It is, therefore, dramatically necessary to seek and develop the novel molecular target for therapy of the patients with ESCC.

Bcl-2 inhibitor of transcription 1 (Bit1) is a kind of mitochondria related protein, encoding 179 amino acid, which is identified through Bcl-2 reporter system [12]. Evolutionarily, Bit1 is verified to be dramatically conserved from bacteria to humans [13]. Data from crystallographic studies have revealed in detail that human Bit1 harbors a peptidyl-tRNA hydrolase domain, which is considered to be implicated in protein translation and folding [14], and thus Bit1 may harbors a large variety of functions involved in the regulation of various biological processes.

In human cells, Bit1 can mediate integrin-dependent survival signals via NF-κB signaling pathway [15], which plays an essential role in the development and progression of many human cancers, suggesting Bit1 is tightly associated with tumor development and progression. Although few studies were reported regarding the connection between Bit1 and tumors, some data from ovarian cancer demonstrated Bit1 overexpression was exhibited in ovarian cancer tissues [16, 17], whereas other evidence showed that Bit1 exerted tumor suppressor in lung cancer [18]. These conflicting data about the different roles of Bit1 in diverse human cancers will reinforce us to further investigate its underlying biological roles in the development and progression of ESCC. Our previous studies revealed that Bit1 overexpression was exhibited in ESCC tissues, and its overexpression was closely correlated with lymphatic metastasis, TNM staging and tumor differentiation [19]. These data suggest that Bit1 plays different roles in various types of cancer, and thereby elucidation of Bit1 function in different tumors will provide a new diagnostic and therapeutic marker for these tumors.

To further verify the underlying functions of Bit1 in ESCC, therefore, in the present study, we examined Bit1 expression in a panel of ESCC cell lines, and investigated the effects of Bit1 knockdown on tumor growth, migration and invasion as well as cell apoptosis in ESCC, and further preliminarily elucidated the possible molecular mechanisms. All data presented herein suggest Bit1 may be a promising molecular target for the therapy of ESCC, and thus intervention of Bit1 may lead to better therapeutic outcomes for the patients with ESCC.

## Results

### Expression of Bit1 in a panel of ESCC cell lines

**Fig. 1:**
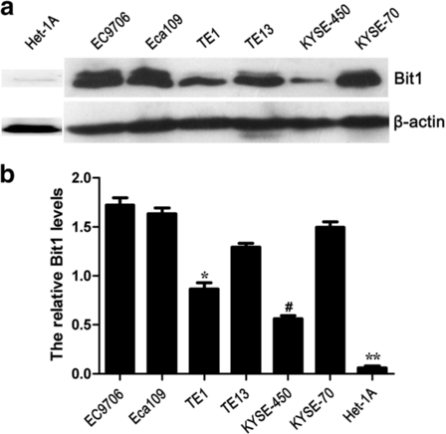
Bit1 protein expression in many different ESCC cell lines and normal esophageal epithelial cell Het-1A. Total protein was extracted from various ESCC cell lines such as EC9706, Eca109, TE1, TE13, KYSE-450 and KYSE-70 as well as Het-1A, and Western blotting was utilized to investigate the Bit1 protein level in ESCC cell lines above. **a** Western blotting assay for Bit1 protein expression in ESCC cell lines including EC9706, Eca109, TE1, TE13, KYSE-450 and KYSE-70 as well as Het-1A, and β-actin was used as internal control. **b** The relative Bit1 protein level was counted by the ratio of Bit1 level to β-actin level, and the results from the three independently repeated experiments, expressed as means ± SD, were investigated by SPSS 17.0 statistical software, *P < 0.05, compared with EC9706, Eca109, TE13, and KYSE-70, #*P* < 0.01, compared with the other ESCC cell lines, **P < 0.01, compared with all of ESCC cell lines

To dissect Bit1 protein level in ESCC, we detected Bit1 protein expression in a series of ESCC cell lines as well as normal esophageal epithelial cell by Western blotting. We found Bit1 protein expression was exhibited at high level in all ESCC cell lines investigated in this study, compared with that in Het-1A (Fig. 1a and b). However, Bit1 level was differentially expressed in these ESCC cell lines, and relative high level was displayed in EC9706, Eca109, TE13 and KYSE-70 cells, the other ESCC cell lines harbored lower Bit1 level, the difference was statistical significance (*P* < 0.05) (Fig. 1a and b). Most notably, expression of Bit1 protein in poorly differentiated KYSE-70 cell line was significantly higher than that in well differentiated KYSE-450, suggesting Bit1 may be positively associated with malignant degree of ESCC. Therefore, to further explore the possible roles of Bit1 in ESCC, EC9706 with the highest Bit1 level was employed to investigate the functions of Bit1.

### Specific shRNA against Bit1 reduces Bit1 protein level in EC9706  and TE1 cells

**Fig. 2:**
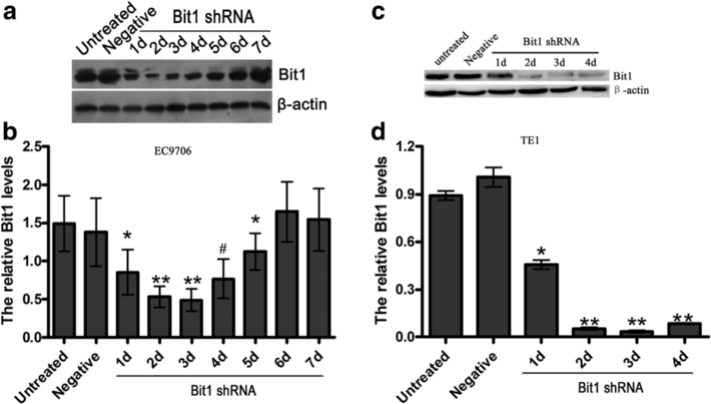
Bit1 shRNA efficiently reduced Bit1 protein level in EC9706 and TE1 cells. Total protein was extracted from EC9706 and TE1 cells untreated and transfected with Bit1 shRNA and negative shRNA at different time points, and Western blotting was used to investigate the Bit1 protein level. **a** Western blotting assay for Bit1 protein level in untreated group, negative group and Bit1 shRNA group at different time points including days 1, 2, 3, 4, 5, 6 and 7 in EC9706 cells. **b** The relative Bit1 protein level was counted by the ratio of Bit1 level to β-actin level in EC9706 cells, and the results from the three independently repeated experiments, expressed as means ± SD, were investigated by SPSS 17.0 statistical software, *P < 0.05, compared with untreated EC9706 cells, negative group, EC9706 cells on days 6 and 7 after transfection with Bit1 shRNA, **P < 0.05, compared with the other groups, # *P* < 0.05, compared with EC9706 cells on day 5. **c** Western blotting assay for Bit1 protein level in untreated group, negative group and Bit1 shRNA group at different time points including days 1, 2, 3 and 4 in TE1 cells. **d** The relative Bit1 protein level was counted by the ratio of Bit1 level to β-actin level in TE1 cells, and the results from the three independently repeated experiments, expressed as means ± SD, were investigated by SPSS 17.0 statistical software, *P < 0.05, compared with untreated EC9706 cells and negative group, **P < 0.05, compared with the other groups

To verify the duration time of Bit1 shRNA in EC9706  and TE1 cells, specific shRNA against Bit1 was used to transfect to EC9706 and TE1 cells, and Bit1 protein expression was detected at days 1, 2, 3, 4, 5, 6, and 7  for EC9706 cells and at days 1, 2, 3 and 4 for TE1 cells by Western blotting. The results demonstrated that compared with EC9706 cells untreated and transfected with negative shRNA, Bit1 protein level was significantly downregulated at day 1 after transfection with specific shRNA against Bit1 (*P* < 0.05). At day 3, Bit1 level reached the lowest value after transfection with Bit1-shRNA. Subsequently, with time progression, Bit1 protein level began to gradually recover the control level, and Bit1 level at days 6 and 7 was similar with that of control group (*P* > 0.05) (Fig. 2a and b). Furthermore, compared with TE1 cells untreated and transfected with negative shRNA, Bit1 protein level was significantly reduced on days 1, 2, 3 and 4, and at day 3, Bit1 level reached the lowest value after transfection with Bit1-shRNA (Fig. 2c and d). These findings suggest that the optimum time of Bit1 shRNA appears at day 3 in both EC9706 and TE1 cells.

### Bit1 knockdown suppresses cell proliferation, migration and invasion in EC9706 and TE1 cells

**Fig. 3:**
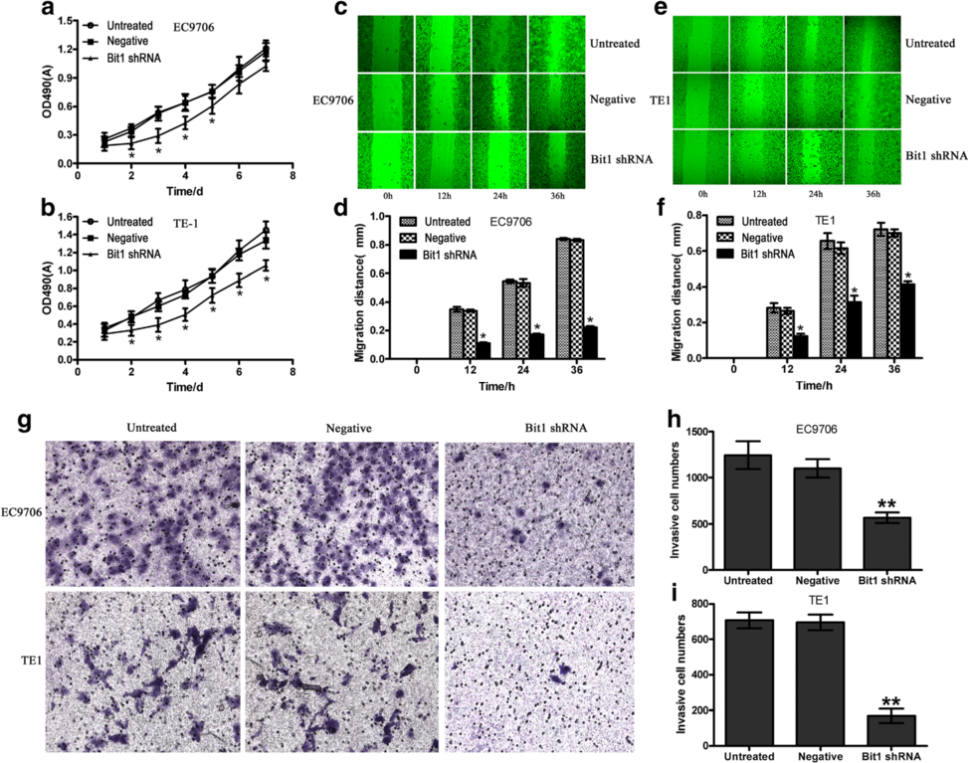
The effects of Bit1 depletion on cell proliferation, migration and invasion abilities in ESCC EC9706 and TE1 cells. **a** and **b** Bit1-shRNA significantly suppressed cell growth on days 2, 3 and 4 in EC9706 and TE1 cells. EC9706 and TE1 cells at a density of 5,000 cells per well were seeded into 96-well culture plates. When cells were cultured for 24 h, and the pSilencer3.1-H1-neo-Bit1-shRNA and pSilencer3.1-H1-neo-negative-shRNA were utilized to transfect EC9706 and TE1 cells. The number of metabolically active cells was assessed by 3- (4, 5-dimethylthiazol-2-yl)-2, 5-diphenyltetrazolium bromide (MTT) assay at each indicated time (1d, 2d, 3d, 4d, 5d, 6d and 7d). **c** and **e** Bit1 knockdown markedly impeded cell migration in EC9706 and TE1 cells. 4 × 10^5^ of EC9706 and TE1 cells were seeded into 6-well culture plates. After transfection with pSilencer3.1-H1-neo-Bit1-shRNA and pSilencer3.1-H1-neo-negative-shRNA for 24 h, in vitro scratch wounds were created by scraping the cell monolayers with a 200 μl sterile pipette tip. The wounded cultures were allowed to grow for 36 h at 37 °C. At 12 h, 24 h and 36 h, photomicrographs were taken at the same position to evaluate the alterations of cell migration ability. **d** and **f** Cell migration was assessed by measuring migration distances from the wound edges. *P < 0.05, compared with untreated and negative group. **g** Bit1 downregulation contributed to the decrease of cell invasion ability in EC9706 and TE1 cells. After transfection with pSilencer3.1-H1-neo-Bit1-shRNA and pSilencer3.1-H1-neo-negative-shRNA for 24 h, EC9706 and TE1 cells (3-5 × 10^4^ per well) were seeded to ECM gel pre-coated, porous upper chamber inserts and allowed to grow at 37 °C in a CO_2_ incubator. The cells that invaded to the bottom surface of the insert were fixed with methanol and stained by 0.5 % crystal violet, and was then subjected to microscopic inspection. **h** and **i** The invasive cell numbers were counted to evaluate the effect of Bit1-shRNA on cell invasion ability in EC9706 and TE1 cells, **P < 0.01, compared with untreated and negative groups

To test whether Bit1 depletion affected cell proliferation, migration and invasion abilities, MTT, Wound healing migration assay and Transwell invasion experiment were utilized to determine the effects of Bit1 knockdown on cell proliferation, migration and invasion abilities, respectively. We found that cell proliferation was significantly suppressed on days 2, 3, 4 and 5 after transfection with Bit1-shRNA in EC9706 cells, compared with untreated group and negative group (*P* < 0.05) (Fig. 3a), which was almost consistent with the data from Bit1 shRNA mediated the alteration of Bit1 protein expression at different time points. However, regarding TE1 cells, Bit1 downregulation also significantly suppressed cell proliferation on days 6 and 7 compared with untreated group and negative group (*P* < 0.05) (Fig. 3b). Stepwise investigation from Wound healing migration assay revealed that migration distance of EC9706 and TE1 cells in Bit1 shRNA group at different time points (12 h, 24 h and 36 h) was all significantly lower than those in untreated group and negative group (*P* < 0.05) (Fig. 3c, d, e and f), suggesting Bit1 shRNA can inhibit cell migration in both EC9706 and TE1 cells. Furthermore, the results from Transwell invasion experiment demonstrated that invasive cell numbers of EC9706 and TE1 cells in Bit1 shRNA group were markedly lower than those in untreated group and negative group (*P* < 0.05) (Fig. 3g, h and i). These findings suggest that Bit1 may exert essential roles in the regulation of cell growth, migration and invasion abilities in ESCC.

### Knockdown of Bit1 expression promotes EC9706 and TE1 cell apoptosis

**Fig. 4:**
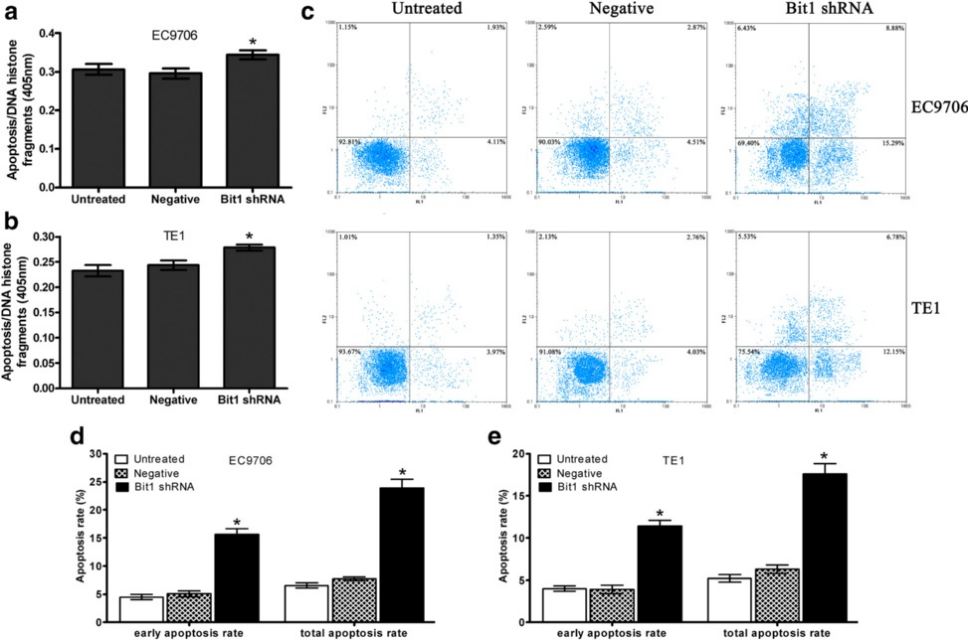
Knockdown of Bit1 expression promotes EC9706 and TE1 cell apoptosis. After transfected with pSilencer3.1-H1-neo-Bit1-shRNA or pSilencer3.1-H1-neo-negative-shRNA for 72 h, cells were subjected to Cell Death ELISA. Three independent experiments were performed. **a** ELISA assay for cell apoptosis in EC9706 cells; **b** ELISA assay for cell apoptosis in TE1 cells; **c** Flow cytometry assay for cell apoptosis in EC9706 and TE1 cells; **d** Three independently repeated experiment for cell apoptosis in EC9706 cells; **e** Three independently repeated experiment for cell apoptosis in TE1 cells; *P < 0.05 as compared to untreated and negative groups

To further confirm the role of Bit1 as an anti-apoptosis factor in ESCC cells, we examined whether knockdown of endogenous Bit1 expression will impact the apoptosis of EC9706  and TE1 cells. We reasoned that EC9706  and TE1 cells underwent apoptosis if ablation of the Bit1 anti-apoptotic pathway may trigger cell apoptosis. Indeed, following a protracted 72 h culture after transfection with pSilencer3.1-H1-neo-Bit1-shRNA, the EC9706 cells eventually showed obvious apoptosis, but there was no statistical difference *(P* > 0.05*)* among different groups (Bit1 shRNA, Negative and Untreated groups) that sample size was calculated according to the previous results using the following equation: ${N}_1={N}_2=2{\left[\frac{\left({Z}_{a/2}+{Z}_{\beta}\right)\sigma }{\delta}\right]}^2$. Where with significance level α = 0.05 and power 1-β = 0.8, we found that the study would therefore need sample size *N*_1_ = *N*_2_ = 10 for the Independent-Samples *T* test. As compared to the EC9706 parental or negative-shRNA treated cells, the Bit1-shRNA transfected cells exhibited increased apoptosis at 72 h (Fig. 4a), and similar results were found in TE1 cells when the sample size N1 = N2 = 15 (Fig. 4b). Furthermore, the results of Flow cytometry demonstrated that the early apoptotic cell numbers and total apoptotic cell numbers of EC9706 and TE1 cells in Bit1 shRNA group were both markedly increased compared with untreated group and negative group (*P* < 0.05) (Fig. 4c, d and e).

### Bit1 downregulation reduced tumorigenicity in EC9706 xenografted nude mice

**Fig. 5:**
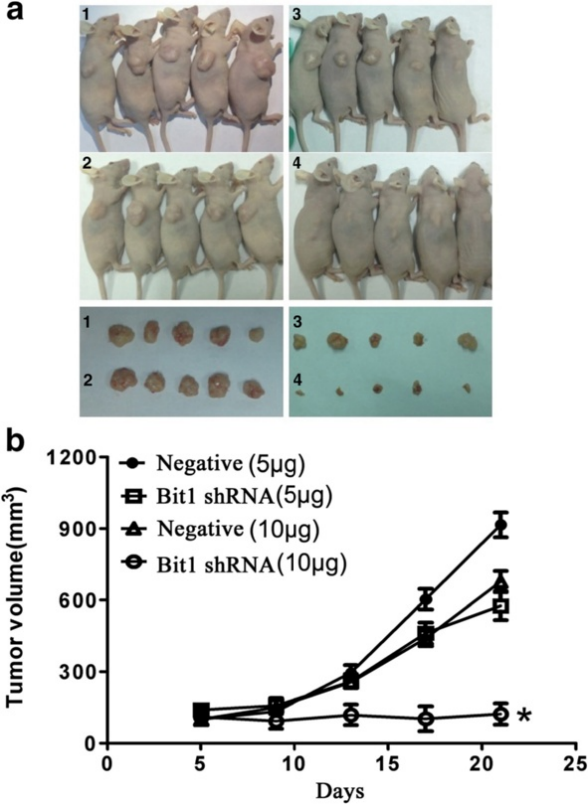
Bit1 shRNA significantly suppressed tumor growth in EC9706 xenografted nude mice. Bit1 shRNA mediated the inhibitory effect on tumor growth in EC9706 xenografted nude mice. EC9706 cells (4 × 10^6^ per nude mouse) were inoculated into the back of right flank of each nude mouse. When tumor volume reached approximately 100 mm^3^ (about on day 5), nude mice were randomly split into four groups: *1*: negative (5 μg), *2*: negative (10 μg), *3*: shRNA (5 μg) and *4*: shRNA (10 μg), and treatment and tumor volume measurement were performed every 4 days. The mice in different groups were injected via intratumor route with the different complexes, which consists of pSilencer3.1-H1-neo-Bit1-shRNA (5 μg and 10 μg) or pSilencer3.1-H1-neo-negative-shRNA (5 μg and 10 μg) and corresponding volume of Lipofectamine 2000. When measurement was terminated, the animals were sacrificed on day 21, and tumor tissues were collected. Finally, tumor growth curve was made to evaluate the effect of Bit1-shRNA on tumor growth. **a** Sacrificed animals and corresponding tumors in different groups. **b** Tumor growth curve in different groups. *P < 0.05, compared with negative groups and shRNA group (5 μg)

Based on cell proliferation inhibition *in vitro* mediated by Bit1 knockdown, we proposed whether decrease of Bit1 level suppressed tumorigenicity in EC9706 xenografted nude mice. In the current study, two doses of pSilencer3.1-H1-neo-Bit1-shRNA or pSilencer3.1-H1-neo-negative-shRNA (5 μg and 10 μg) were employed to treat the tumors in EC9706 xenografted nude mice model. We found that compared with negative group, 10 μg of pSilencer3.1-H1-neo-Bit1-shRNA significantly suppressed tumor growth (*P* < 0.05) (Fig. 5a and b), but 5 μg of pSilencer3.1-H1-neo-Bit1-shRNA didn’t inhibit tumor growth (*P* > 0.05) (Fig. 5a and b), suggesting Bit1-shRNA mediated inhibitory effect on tumor growth displays in a dose-dependent manner.

### Bit1-shRNA reduced the levels of Bit1, Bcl-2 and MMP-2 as well as induced cell apoptosis in EC9706 xenografted nude mice

**Fig. 6:**
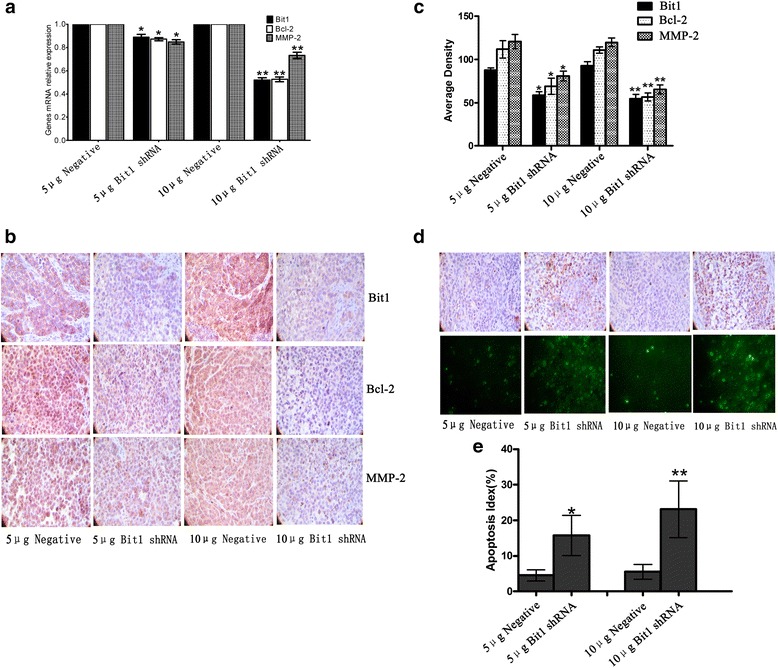
Bit1-shRNA triggered the decreases of Bit1, Bcl-2 and MMP-2 expressions as well as cell apoptosis in xenografted nude mice. When the measurement of tumor volume was terminated, tumor tissues were harvested, and real-time qPCR and Western blotting were utilized to detect the expressions of Bit1, Bcl-2 and MMP-2 mRNAs and proteins. **a** Real-time qPCR investigation for expressions of Bit1, Bcl-2 and MMP-2 mRNAs in different doses Bit1 shRNA or negative shRNA groups. **b** Immunohistochemistry assay for expressions of Bit1, Bcl-2 and MMP-2 proteins in different doses Bit1 shRNA or negative shRNA groups. **c** Average density assay of Bit1, Bcl-2 and MMP-2 protein levels in different doses Bit1 shRNA or negative shRNA groups. **d** TUNEL assay for cell apoptosis in different doses Bit1 shRNA or negative shRNA groups. **e** Apoptotic index was counted according to apoptotic cell number under 5 randomly selected fields. *P < 0.05, compared with 5 μg negative group, **P < 0.01, compared with 10 μg negative group

To further delineate the expressions of Bcl-2 and MMP-2 proteins closely related to Bit1, the sacrificed nude mice tumor tissues were carefully analyzed by qRT-PCR, Immunohistochemistry and In Situ Apoptosis Detection assays as described above. The results revealed that Bit1, Bcl-2 and MMP-2 mRNA level in xenografts tissues of 5 μg-shRNA treated group was significantly lower than those in 5 μg-negative treated group (*P* < 0.05), similarly, the mRNA levels of the three genes in 10 μg-shRNA treated group were also markedly less than those in 10 μg-negative treated group (*P* < 0.01) (Fig. 6a). In addition, the IOD values of Immunohistochemistry related expressions of Bit1, Bcl-2 and MMP-2 proteins were in line with the results of qRT-PCR. Briefly, expressions of Bit1, Bcl-2 and MMP-2 proteins in 5 μg-shRNA group (58.44 ± 4.17, 68.89 ± 9.43 and 80.94 ± 5.76) were obviously lower than those in 5 μg-negative group (87.57 ± 2.51, 111.83 ± 10.08 and 120.64 ± 8.29), similarly, their expressions in 10 μg shRNA-group (54.47 ± 5.27, 56.62 ± 4.50 and 65.50 ± 5.33) were also remarkably reduced compared with 10 μg-negative group (92.97 ± 4.72, 110.91 ± 3.59 and 119.84 ± 4.94) (Fig. 6b and c). Besides, in 5 μg-shRNA, 5 μg-negative, 10 μg-shRNA and 10 μg-negative groups, the numbers of apoptotic cells were 45/1000, 157/1000, 55/1000 and 231/1000, respectively, and the differences were statistical significance (*P* < 0.05) (Fig. 6d and e). These data presented herein suggest that Bit1 downregulation may be tightly associated with the reduces of Bcl-2 and MMP-2 protein levels, and thus results in the alterations of cell apoptosis, migration and invasion of EC9706 cells.

### Bit1 downregulation altered gene expression profiles in EC9706 cells

**Fig. 7:**
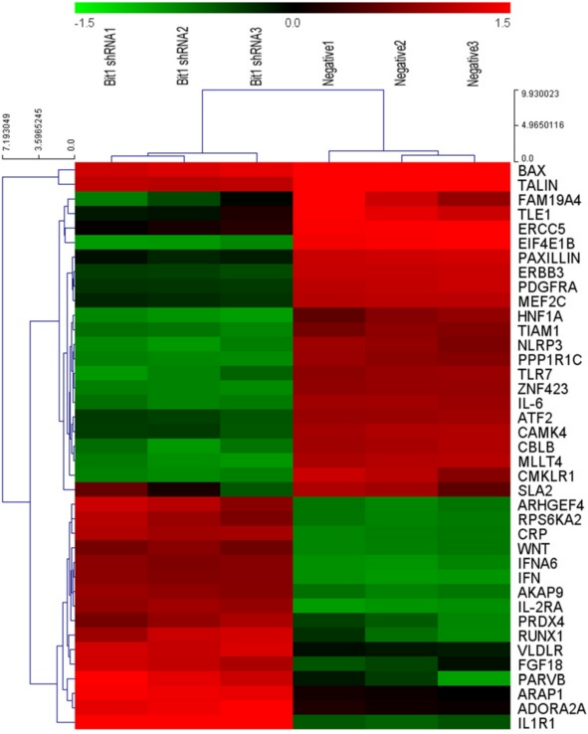
Gene chip assay for differently expressed genes in EC9706 cells treatment with Bit1 shRNA or negative shRNA. EC9706 cells were transfected with Bit1-shRNA or negative shRNA, and cells were subjected to gene chip assay. Data were annotated using the SAS Analysis System and filtered by signal intensity and detection call. Gene expression fold changes were compared between Bit1-shRNA treated and negative-shRNA treated cells. *P < 0.05, differently expressed genes between two groups were considered as statistical significance

To further dissect the possible molecular mechanisms mediated by Bit1 downregulation, microarray was utilized to identify differently expressed genes in Bit1 shRNA group and negative group. According to method of multiplicity, 143 differently expressed genes were screened out in Bit1 shRNA group and negative control group in EC9706 cells, in which 79 genes were upregulated and 64 genes were downregulated (*P* < 0.05). 143 differently expressed genes were submitted to DAVID website, and 113 genes were recognized by DAVID database. In addition, functional annotation using the Gene Ontology (GO) terms demonstrated that 24 genes was appeared in GO function annotation clusters, and its functional type was implicated in cell adhesion, extracellular matrix joint and biological adhesion, ect. These genes were tightly associated with several signaling pathways, including Notch signaling pathway,NOD-like receptor signaling pathway,MAPK signaling pathway, Focal adhesion,PI3K-Akt signaling pathway, etc. In functional classification and signaling pathways above, 16 upregulated genes (AKAP9, ARHGEF4, RUNX1, FGF18, PARVB, PRDX4, RPS6KA2, ADORA2A, IL1R1, CRP, WNT, IL-2RA, IFN, ARAP1, IFNA6 and VLDLR) and 23 downregulated genes (CMKLR1, SLA2, HNF1A, PPP1R1C, ZNF423, FAM19A4, TLE1, NLRP3, PAXILLIN, BAX, CBLB, MLLT4, ERBB3, MEF2C, ATF2, TALIN, PDGFRA, TIAM1, CAMK4, EIF4E1B, ERCC5, TLR7 and IL-6) were selected for future investigation (Fig. 7). These findings suggest that Bit1 may be involved in multiple different molecular mechanisms in ESCC.

### Bit1 interacted with FAK protein as well as Bit1 downregulation evoked the decrease of FAK and Paxillin expressions in EC9706 cells

**Fig. 8:**
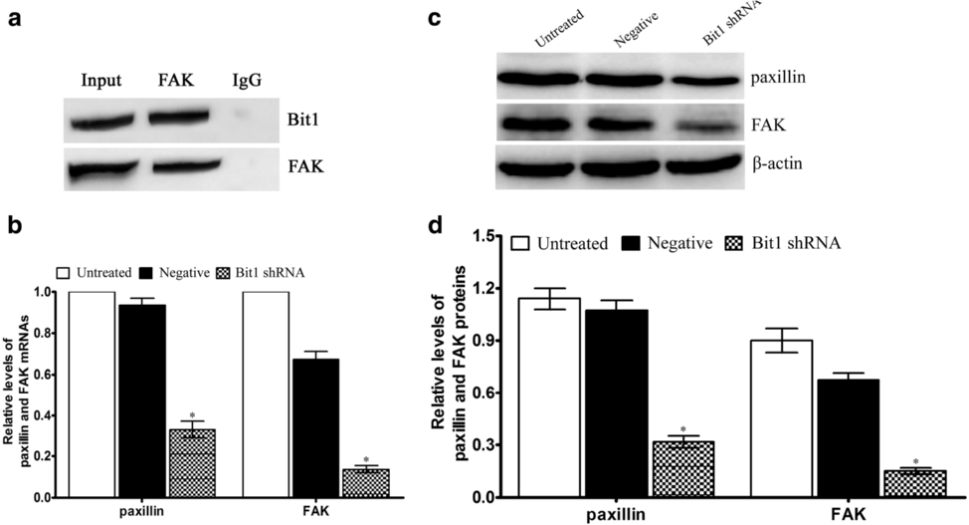
Bit1 interacted with FAK protein as well as Bit1 downregulation contributed to the suppression of FAK-paxillin signaling pathway. **a** Bit1 forms a complex with FAK protein in either Bit1 or FAK immunoprecipitation (IP). **b** Real-time qPCR investigation for the relative levels of paxillin and FAK mRNAs in different treatment EC9706 cells, and GAPDH was utilized as internal control, the relative levels of paxillin and FAK mRNAs were counted according to 2^-△△Ct^. **c** Western blotting assay for the expressions of paxillin and FAK proteins in different treatment EC9706 cells, and β-actin was utilized as internal control. **d** Relative levels of paxillin and FAK proteins were counted according to the ratio of interest protein to β-actin. *P < 0.05, compared with untreated group and negative group

Study demonstrated that Bit1 and FAK were in a complex in adherent cells [15], we put forward whether Bit1 could interact with FAK in EC9706 cells. We found that on both substrates, FAK were detected in Bit1 immunoprecipitations, meanwhile, Bit1 was detected in FAK immunoprecipitations (Fig. 8a) It is well documented that FAK and Paxillin is tightly associated with tumor progression, migration, invasion and metastasis in many tumors [20–24]. Therefore, to further verify the validation of gene chip, real-time qPCR and Western blotting was utilized to detect the expressions of paxillin and FAK in Bit1 shRNA group. We found that Bit1 shRNA significantly donwregulated paxillin mRNA and protein expressions, compared with untreated group and negative group (*P* < 0.05) (Fig. 8b, c and d), which was consistent with gene chip results, coupled with reduced FAK expression (*P* < 0.05) (Fig. 8b, c and d). These findings suggest that Bit1 downregulation mediated reduced invasion ability may be achieved via FAK-paxillin signaling pathway.

## Discussion

Increasing evidence has demonstrated that mitochondria dysfunction leads to many diseases [25–28], such as Alzheimer, Parkinson, tumor, etc., which is tightly associated with aberrant alterations of gene expression profiles. Bit1, as a mitochondria protein, also was verified to be implicated in tumor development and progression in many documented publications [18, 29–31]. Our previous studies demonstrated that Bit1 expression was significantly higher than that in normal esophageal tissues [19], which was further supported by our current study in a panel of ESCC cell lines. Interestingly, Bit1 expression in poorly-differentiated ESCC cell lines was higher than that in well-differentiated ESCC cell lines, suggesting Bit1 may function as oncogene in ESCC. Overall, these findings suggest that Bit1 may play an essential role in the development and progression of ESCC.

It is well documented that balance between tumor oncogene and suppressor play predominant roles in the development and progression of a variety of human tumors. Bit1, as an oncogene or a tumor suppressor, seems to be in a context-dependent manner through many different molecular mechanisms. Yao X et al. found that reintroduction of Bit1 was sufficient to suppress the anchorage-independent growth; conversely, Bit1 downregulation significantly enhances anchorage-independent growth in A549 cells and further investigation revealed that the Bit1-depletion cells displayed significantly enhanced tumorigenecity in vivo [18], implying Bit1 functions as tumor suppressor in lung carcinoma. Conversely, Bit1 depletion contributed to cell growth suppression in cervical cancer cell line HeLa, compared with control cells [15]. To test whether Bit1 downregulation affected cell growth in ESCC, MTT assay and nude mice xenografts were utilized to investigate the effects of Bit1 knockdown on cell growth in ESCC, we found that Bit1 downregulation markedly suppressed cell growth in vitro and in vivo, implying Bit1 functions tumor suppressor in ESCC.

Invasion and metastasis are one of the typical hallmarks of tumors. The investigation from Karmali PP and colleagues demonstrated that metastatic numbers and foci in Bit1-depletion B16F1 cells were significantly increased compared with control B16F1 cells, whereas Bit1 overexpression by exogenous introduction reduced the formation of lung metastatic colonies [32], implying Bit1 functions as the suppressor of metastasis in melanoma. Contrary to this result, in the current study, we found Bit1 downregulation contributed to the decreases of migration and invasion abilities of ESCC EC9706 and TE1 cells. These findings suggest that the role exerted by Bit1 in invasion and metastasis may be associated with tumor types, however, its precise molecular mechanisms involved in invasion and metastasis of ESCC remains to be elucidated in future.

In previous study, Bit1 was verified to be a critical apoptosis regulator through manipulation of Bcl-2 expression [12]. Several studies have demonstrated that Bcl-2 is tightly associated with tumor development and progression [33–35], and thus may be an underlying molecular biomarker. It is well documented that MMP-2 is implicated in tumor invasion and metastasis, which is also confirmed in some publications [36–38]. Therefore, to further confirm the role of Bit1 in EC9706 cells xenografted nude mice, we found that Bit1 downregulation mediated by shRNA significantly decreased the levels of Bcl-2 and MMP-2 mRNA and proteins in EC9706 cells xenografted nude mice, compared to negative groups, coupled with the increases of apoptotic index. These data indicate that the alterations of cell growth, migration and invasion triggered by Bit1 depletion is tightly involved in the regulation of Bcl-2 and MMP-2, however, precise molecular mechanisms related to these changes above is under investigation.

Understanding the molecular mechanisms of Bit1 involved in development and progression of tumors is crucial and helps to accelerate the development of drugs that target Bit1 or its related signaling pathways. To further clarify the signaling pathway mediated by Bit1 downregulation, we performed array assay by gene chip, we found that Bit1 downregulation triggered 23 genes downregulation and 16 genes upregulation in EC9706 cells, which was implicated in cell apoptosis, cell adhesion, invasion and metastasis, etc., suggesting Bit1 may be an important regulator in cell invasion and metastasis of ESCC. Currently, increasing evidence has demonstrated that paxillin is highly expressed in many tumors, and is tightly associated with development, progression, invasion, metastasis and poor prognosis of a multiple of human tumors [39–45]. Furthermore, FAK-paxillin signaling pathway participates in the processes of tumor invasion and metastasis via different molecular mechanisms in many human tumors [46–48], and thus may be a potential predictor for metastasis of tumors. To establish the connections of Bit1 and FAK-paxillin signaling pathway, immunoprecipitation was employed to verify the interaction of Bit1 and FAK proteins in EC9706 cells. We found Bit1 intrinsically interacted with FAK protein in EC9706 cells. To further understand the underlying mechanisms of Bit1 downregualtion mediated the alterations of migration and invasion, and verify the validation of paxillin expression obtained by gene chip in EC9706 cells, we detected the expressions of paxillin and its related protein FAK by Real-time qPCR and Western blotting. The results revealed that paxilllin and FAK expressions were markedly decreased, compared with untreated group and negative control group, implying Bit1 functions as invasion and metastasis- regulator through FAK-paxillin signaling pathway, however, precise molecular mechanisms mediated by Bit1 downregulation remain under investigation. Based on the results above, we put forward to a novel pathway Bit1-FAK-paxillin, which participates in the invasion and metastasis of ESCC, and manipulation of the signaling pathway may be a potentially molecular target in future.

## Conclusion

Collectively, our data suggest that Bit1 expression is exhibited at the high level in ESCC cell lines, and its expression in poorly-differentiated ESCC cell lines is significantly higher than that in well-differentiated ESCC cell lines. Bit1 downregulation significantly suppresses cell growth in vitro and in vivo, and reduces the migration and invasion abilities in ESCC cells, which may be tightly associated with decreases of Bcl-2 and MMP-2 expressions. Combined with gene chip results, stepwise investigation indicates that Bit1 intrinsically interacted with FAK protein in EC9706 cells, and Bit1 downregulation significantly reduces paxillin and FAK expression. Future directions will be to develop the Bit1 as novel molecular target for the patients with ESCC, which may be implicated in large numbers of molecular regulation mechanisms of Bit1 in ESCC.

## Methods

### Cell lines and culture conditions

The human ESCC cell lines EC9706, Eca109, TE1, TE13, KYES-450 and KYSE-70 were all purchased from the Tumor Cell Bank of Chinese Academy of Sciences, and both maintained in Roswell Park Memorial Institute (RPMI) 1640 media supplemented with 10 % Fetal bovine serum (FBS), 1 % L-Glutamine and 1 % Penicillin-Streptomycin (Solarbio Life Technologies, Beijing, China). Normal esophageal epithelial cell Het-1A immortalized by introducing plasmid pRSV-T with RSV-LTR promoter and SV40 T antigen [49] was purchased from the American Type Culture Collection (Manassas, VA, USA), which was also maintained under the culture conditions above. All of the cells were cultured at 37 °C under 5 % humidified CO_2_ enriched atmosphere.

### Protein extraction and Western blotting

Cells were washed twice with ice-cold PBS, and collected cells were lysed in buffer (Beyotime Institute of Biotechnology, Shanghai, China). Protein concentration was determined using Bradford reagent (Sigma-Aldrich, San Luis, MO, USA) according to standard precedures. Total protein (0.4 mg) was added to 5× protein sample buffer, heated at 100 °C for 2 min and separated by SDS-PAGE with 3 μl of pre-stained protein molecular weight marker (Fermentas Life Sciences, Burlington, ON, Canada) as a standard, which was transferred to a polyvinylidene fluoride (PVDF) membrane and incubated with the following respective primary antibodies including anti-Bit1 (1:10000), anti-FAK (1: 1000), anti-paxillin (1:200), and control anti-β-actin (1:500) overnight at 4 °C, followed by secondary antibodies (goat anti mouse, 1:5000, goat anti rabbit, 1:2000) conjugated with horseradish peroxidase. Membranes were developed using the ECL detection system (Beyotime Institute of Biotechnology, Shanghai, China). Quantification of Western blotting was performed using Quantity-One software (NIH) to determine the relative protein level, and the results were from at least three independently repeated experiments.

### Quantitative real-time RT-PCR

Quantitative real-time RT-PCR (real-time qPCR) assay was performed as our previous report [19]. Briefly, total RNA was extracted from 100 mg of the mice tumors or cells by using TRIzol reagent (Invitrogen, USA). The cDNA was prepared using a reverse transcription reagent kit (TaKaRa Company, Dalian, China) according to the manufacturer's instructions. Target genes were amplified using ABI 7500 Real-Time PCR System (USA) with the following specific primers: Bit1, 5’-AGAGGTAGCTCACGCGATAGAA-3’ (forward) and 5’-GCATCCCAAAGCATA CTCGAA-3’ (reverse) (product length, 196 bp); Bcl-2, 5′-CAGCTGCACCTGACGCC CTT-3’ (forward) and 5’-GCCTCCGTTATCCTGGATCC-3’ (reverse) (product length, 230 bp); MMP-2, 5’-CTCCTGACATTGACCTTGGC -3’ (forward) and 5’-CCTCGCT CCAGGGTGCTGGC-3’ (reverse) (product length, 310 bp); FAK, 5’-AGTAAAATCC AGCCAGCCCC-3’ (forward) and 5’-GACATACTGCTGGGCCAGTT-3’ (reverse) (product length, 207 bp); paxillin, 5’-CTGTCGGATTTCAAGTTCATGGC-3’ (forward) and 5’-TGGGTGCAGACGAAG TGC-3’(reverse) (product length, 239 bp) as well as GAPDH, 5’-GCACCGTCAAGGCTGAGAAC-3’ (forward) and 5’-TGGTGAA GACG CCAGTGGA-3’ (reverse) (product length, 138 bp); GAPDH was utilized as normalization. Each sample was tested in triplicate, and the gene relative levels were normalized using the 2^−ΔΔCt^ method according to a previous description [50].

### Liposome mediated cell transfection assay

EC9706  and TE1 cells were seeded in a 6-well plate at a density of 4 × 10^5^ for 24 h before transfection. The pSilencer3.1-H1-neo-negative-shRNA and pSilencer3.1-H1-neo-Bit1 -shRNA plasmids were generated as described previously [31]. Experiments were divided into three groups: untreated group (transfection with Lipofectamine 2000), negative group (transfection with pSilencer3.1-H1-neo-negative-shRNA), and Bit1 shRNA group (transfection with pSilencer3.1-H1-neo-Bit1-shRNA). Transfection was carried out according to the manufacturer’s instructions using pSilencer3.1-H1-neo-Bit1-shRNA or pSilencer3.1-H1-neo-negative-shRNA plasmids at final quantifications of 2-4 μg, and untreated group was transfected with Lipofectamine 2000 reagent (Invitrogen, USA) alone as control. Cells were harvested and subjected to immunoblotting, migration, transwell, ELISA, or Microarray assays as described below 72 h after transfection.

### Cell proliferation

For proliferation assay, cells were plated in a volume of 150 μl at a density of 5,000 cells per well in 96-well plates, which was allowed grow for 24 h and then transfected with the pSilencer3.1-H1-neo-Bit1-shRNAor pSilencer3.1-H1-neo-negative-shRNA. At each indicated time point (1d, 2d, 3d, 4d, 5d, 6d and 7d for EC9706 cells and 1d, 2d, 3d and 4d for TE1 cells), the numbers of metabolically active cells were assessed by 3- (4, 5-dimethylthiazol-2-yl)-2, 5-diphenyltetrazolium bromide (MTT) assay. Briefly, MTT reagent (Sigma, USA) was dissolved in sterile PBS at 5 mg/ml. 20 μl of this solution was added to each well in a 96-well plate (1:5 dilution). The plate was then incubated for 4 h at 37 °C in 5 % humidified CO_2_ enriched atmosphere. Afterwards, the medium was gently aspirated away and the MTT precipitate was dissolved in 100 μl of a 50 % MeOH-50 % DMSO solution. The precipitate was allowed to dissolve at room temperature for 10 min with gentle shaking. Absorbance (A) was measured at a wavelength of 490 nm using a Microplate Reader (BIO-TEK, Winooski, USA).

### Cell migration experiment

Migration of ESCC EC9706 and TE1 cells was investigated by Wound healing migration assay. Viable cells were plated at 4 × 10^5^ cells per well in 6-well culture plates using growth media containing 10 % FBS. After transfection with pSilencer3.1-H1-neo-Bit1-shRNA and pSilencer3.1-H1-neo–negative-shRNA vectors for 24 h, *in vitro* scratch wounds were created by scraping the cell monolayers with a 200 μl sterile pipette tip. After washing away suspended cells, photomicrograph was taken immediately (time 0 h) with an inverted microscope equipped with a digital camera, and the wounded cultures were allowed to grow for 36 h at 37 °C. At 12 h, 24 h, 36 h, photomicrographs were taken at the same position, respectively. Migrations at least three independently repeated experiments were quantified by measuring distances from the wound edges.

### Cell invasion assay

To determine whether the invasion ability of ESCC EC9706 and TE1 cells was mediated by Bit1 shRNA. Transwell invasion assay was performed as Corning’s Transwell chambers (24-well plate, 6.5 mm in diameter with 8.0 μm pores) with 100 μl of Matrigel basement membrane matrix (BD Bioscience, Bedford, MA) per well and solidified at 37 °C for 30 min. Briefly, after transfection with pSilencer3.1-H1-neo-Bit1-shRNA or pSilencer3.1-H1-neo-negative-shRNA for 24 h, cells (3-5 × 10^4^ per well) were seeded into ECM gel pre-coated, porous upper chamber inserts and allowed to invade overnight at 37 °C in a CO_2_ incubator. Subsequently, the insert was washed with PBS and the cells on the top surface of the insert were removed by wiping with a cotton swab. The cells that invaded the bottom surface of the insert were fixed with methanol and stained by 0.5 % crystal violet and then subjected to microscopic inspection. All fields were chosen and the numbers of penetrated cells were counted at 200× magnification. All data were calculated based on triplicate experiments.

### Histone/DNA fragment ELISA

Exponentially growing EC9706 and TE1 cells were plated in sterile petri dishes and transfected with pSilencer3.1-H1-neo-Bit1-shRNA or pSilencer3.1-H1-neo-negative -shRNA. Cytosolic fractions of 5 × 10^4^ cells per group served as an antigen source in a sandwich ELISA using primary anti-histone antibody-coated microplate and a secondary peroxidase-conjugated anti-DNA antibody. The photometric immunoassay for histone-associated DNA fragments was executed according to the manufacturer’s instructions and absorbance (A) value was measured at 405 nm using a Microplate Reader (BIO-TEK, Winooski, USA). A higher A value was correlated with increased apoptosis. All data were calculated based on triplicate experiments.

### Immunoprecipitations (IP)

EC9706 cells were lysed for 30 min on ice with immunoprecipitations (IP) buffer (Pierce, Rockford, IL). The lysates were centrifuged at 12,000 g for 10 min at 4 °C. The cell lysates (500 μg) was mixed with 5 μg of antibodies against Bit1 or FAK, respectively. Subsequently, immune complexes were collected with elution buffer at 3000 g centrifugation for 1 min at 4 °C according to manufacturer’s protocol. Finally, the samples were submitted to immunoblotting assay.

### *In vivo* experiment

All procedures were done according to protocols approved by the Institutional Committee for Use and Care of Laboratory Animals of Zhengzhou University. Female BALB/c nude mice (4–6 weeks old) were purchased from the Beijing Weitong Lihua Experimental Animal Technical Co., Ltd. To observe the effect of Bit1 on tumor growth *in vivo*, EC9706 cells (4 × 10^6^) were subcutaneously implanted into one flank in each of 20 nude mice. Once the tumor volumes reached approximate 100 mm^3^ (length × width^2^/2, about on day 5), the mice were randomly divided into four groups. The different complexes, which consisted of pSilencer3.1-H1-neo-Bit1-shRNA (5 μg and 10 μg) or pSilencer3.1-H1-neo-negative-shRNA (5 μg and 10 μg) and corresponding volume of Lipofectamine 2000, were injected to nude mice via intratumor route every 4 days (injection on days 5, 9, 13, 17, respectively). The mice were sacrificed on day 21, then the tumor tissues were harvested and qRT-PCR, TUNEL and immunohistochemistry assays were performed as described below.

### Immunohistochemistry

Immunohistochemistry was performed on 4 mm thick paraffin-embedded tissue sections as our previous description [19]. Briefly, the processed sections of Bit1 protein were incubated with the same antibody as the Western blotting, while Bcl-2 and MMP-2 protein were used anti-Bcl-2 (1:2000) and anti-MMP-2(1:1000), respectively, and PBS instead of Bit1, Bcl-2, and MMP-2 primary antibodies was used as a negative control. The DAB Detection Kit was used to develop staining signal according to the protocols provided for the Ventana Molecular Discovery System (Illkirch, France). The slides were counterstained with haematoxylin. All sections were investigated by light microscopy. The staining evaluation was performed by calculating the IOD (integrate optical density) using the software of Biosens Digital Imaging System V1.6 (Shanghai, China) at 400× magnification. All of the normal and neoplastic cells that both exhibited cytoplasmic immunoreactivity with clearly brown-yellow were regarded as Bit1, Bcl-2, and MMP-2 positive staining. A scoring system for Bit1, Bcl-2 and MMP-2 positive staining was carried out by the software automatically.

### In Situ Apoptosis Detection

Detection of apoptotic cells in tumor sections was performed using the DeadEnd Colorimetric TUNEL System (Promega) following the manufacturer’s instructions. Briefly, sections were deparaffinized, rehydrated, and incubated with proteinase K for 20 min at room temperature. After washing with PBS, the sections were incubated with a working concentration of recombinant Terminal Deoxynucleotidyl Transferase (rTdT) at 37 °C for 1 h in wet and dark atmosphere. The sections were washed with PBS and immersed in 0.3 % hydrogen peroxide to block endogenous peroxidase activity. The sections were subsequently washed with PBS and incubated with the 50 μl streptavidin-HRP working solution at 37 °C for 30 min. Finally, the resulting dark brown signal was visualized with Diaminobenzidine (DAB) as chromogen. Negative control (label solution contained no terminal deoxynucleotidyl transferase in TUNEL reaction mixture) and positive control (incubation of permeabilized cells with 100 μl DNAse 1 buffer for 10 min at 15-25 °C, prior to labeling procedures) were also performed as internal controls.

### RNA isolation and whole-genome gene expression profiling of EC9706 cells

EC9706 cells were harvested 72 h after transfection with pSilencer3.1-H1 -neo-Bit1-shRNA or pSilencer3.1-H1-neo-negative-shRNA. Approximate 1 × 10^6^ cells from each sample were subjected to gene microarray assay. Total RNA was extracted using TRIzol reagent (Invitrogen, USA) following the manufacturer’s instructions and analyzed with Agilent SurePrint G3 Human GE 8 × 60 K Microarray (Agilent technologies) by the Shanghai Biochip Company (Shanghai, China). Data were annotated using the SAS Analysis System and filtered by signal intensity and detection call [present (P), marginal (M), or absent (A)]. Gene expression fold changes were compared between Bit1-shRNA treated and negative-shRNA treated cells. Microarray data were deposited in the NCBI GEO database (http://www.ncbi.nlm.nih.gov/geo/query/acc.cgi?acc=GSE78813).

### Statistical analysis

SPSS 17.0 statistical software package (SPSS, Inc., Chicago, IL, USA) was used for statistical treatment. For the measurement data, data were presented as mean ± SD. Differences among the groups were determined using one-way ANOVA, and then multiple comparisons between groups were performed using LSD *t*-test. For the count data, rank sum test was used in the comparisons between groups. A value of *P* less than 0.05 was considered as statistically significant.
